# BDNF Overexpression in the Ventral Hippocampus Promotes Antidepressant- and Anxiolytic-Like Activity in Serotonin Transporter Knockout Rats

**DOI:** 10.3390/ijms22095040

**Published:** 2021-05-10

**Authors:** Danielle M. Diniz, Francesca Calabrese, Paola Brivio, Marco A. Riva, Joanes Grandjean, Judith R. Homberg

**Affiliations:** 1 Centre for Neuroscience, Department of Cognitive Neuroscience, Donders Institute for Brain, Cognition, and Behaviour, Radboud University Nijmegen Medical Centre, 6525 EN Nijmegen, The Netherlands; danielle91diniz@gmail.com (D.M.D.); Joanes.Grandjean@radboudumc.nl (J.G.); 2 Department of Pharmacological and Biomolecular Sciences, Universita’ degli Studi di Milano, 20133 Milano, Italy; francesca.calabrese@unimi.it (F.C.); paola.brivio@unimi.it (P.B.); m.riva@unimi.it (M.A.R.); 3 Biological Psychiatry Laboratory, IRCCS Istituto Centro San Giovanni di Dio Fatebenefratelli, 25125 Brescia, Italy; 4 Department of Radiology and Nuclear Medicine, Donders Institute, Radboud University Medical Centre, 6500 HB Nijmegen, The Netherlands

**Keywords:** BDNF, serotonin knockout rats, hippocampus, depression, anxiety

## Abstract

BDNF plays a pivotal role in neuroplasticity events, vulnerability and resilience to stress-related disorders, being decreased in depressive patients and increased after antidepressant treatment. BDNF was found to be reduced in patients carrying the human polymorphism in the serotonin transporter promoter region (5-HTTLPR). The serotonin knockout rat (SERT^−/−^) is one of the animal models used to investigate the underlying molecular mechanisms of depression in humans. They present decreased BDNF levels, and anxiety- and depression-like behavior. To investigate whether upregulating BDNF would ameliorate the phenotype of SERT^−/−^ rats, we overexpressed BDNF locally into the ventral hippocampus and submitted the animals to behavioral testing. The results showed that BDNF overexpression in the vHIP of SERT^−/−^ rats promoted higher sucrose preference and sucrose intake; on the first day of the sucrose consumption test it decreased immobility time in the forced swim test and increased the time spent in the center of a novel environment. Furthermore, BDNF overexpression altered social behavior in SERT^−/−^ rats, which presented increased passive contact with test partner and decreased solitary behavior. Finally, it promoted decrease in plasma corticosterone levels 60 min after restraint stress. In conclusion, modulation of BDNF IV levels in the vHIP of SERT^−/−^ rats led to a positive behavioral outcome placing BDNF upregulation in the vHIP as a potential target to new therapeutic approaches to improve depressive symptoms.

## 1. Introduction

An essential part of our daily performance has to do with our capability to adjust and respond to unforeseen events. Adaptation to unexpected environmental changes relies on our ability to develop resilience and display behavioral flexibility. This flexibility is a result of several mechanisms occurring in the brain that are generally called plasticity. Brain plasticity or neuroplasticity can be defined as the adjustments the nervous system undergoes in response to diverse stimuli to achieve reorganization at the structural, functional or cellular connectivity level [1]. Considering this, it is expected that alterations in the brain’s ability to develop neuronal plasticity can lead to maladaptation and, consequently, to the development of neuropsychiatric disorders [2]. Many molecular networks might be involved in neuroplasticity, including the serotonin and the brain-derived neurotrophic factor (BDNF) systems. Serotonin is a brain-wide distributed monoamine, and its decrease has been connected to the occurrence of depression [3]. The levels of serotonin in the synaptic cleft are regulated by the serotonin reuptake transporter (SERT), and to restore serotonin levels in depressive subjects, drugs targeting SERT, known as selective serotonin-reuptake inhibitors (SSRI), are considered the first-line treatment for depressive disorders [4,5]. While SSRIs very quickly generate a blockade of SERT in vitro, the actual clinic therapeutic effect may take several weeks [6]. This delay suggests that the molecular mechanisms underlying depression might be more complex, requiring perhaps secondary changes in structural, functional or cellular connectivity, thus requiring neuronal plasticity [2,7].

BDNF, a neuropeptide from the neurotrophin family, plays a central role in neuroplasticity [8]. Several studies confirmed that BDNF contributes to the brain plasticity through its positive influence in neurogenesis, cell survival, synapse formation and plasticity [8,9,10]. BDNF gained attention in the study of mood disorders because data from post mortem brain tissues or serum of depressive patients indicated low levels of BDNF, which were normalized following antidepressant treatment [11,12]. Accordingly, BDNF has been associated with the mechanism of action of antidepressants [13,14,15]. Therefore, the relation between the serotonin system and the BDNF system in connection with the antidepressant therapeutic effect seems to overlap.

The human SERT, encoded by the SLC6A4 gene, presents a functional polymorphism in the promoter region (5-HTTLPR), generating a short and a long allele variant of the serotonin transporter [16]. The short allelic variant induces a decrease in the SERT transcription in comparison to the long allelic variant [17], and it is linked to increased risk of depression and suicidal behavior [18,19,20,21]. Additionally, the short allele variant appears to be associated with insufficient response to SSRIs [22]. Moreover, the short allelic variant has been related to decreased BDNF mRNA levels in white blood cells and serum from healthy 5-HTTLPR patients [23,24,25]. Likewise, rodents lacking SERT (SERT^−/−^ animals) display a significant BDNF mRNA and protein downregulation, especially in the hippocampus and prefrontal cortex [25,26,27]. Additionally, SERT^−/−^ rats and mice present anxiety- and depression-related phenotypes [28,29].

The hippocampus plays a crucial role in depression. The hippocampus modulates emotional processing, memory and learning and controls glucocorticoid secretion by the hypothalamic-pituitary-adrenal axis (HPA-axis), making this area susceptible to the effects of stress [30]. Stress and other negative stimuli can change hippocampal plasticity, increasing the risk of depression [31]. In fact, depressive disorder is significantly associated with hippocampal atrophy [32,33,34,35]. Furthermore, several lines of research have shown that BNDF and its high-affinity receptor (TrkB) are decreased in the hippocampus of post mortem tissue from suicidal or depressed patients [36,37,38,39]. Importantly, while impaired hippocampal neurogenesis can lead to depression [40], studies have demonstrated that the upregulation of BDNF levels stimulate hippocampal neurogenesis [41,42]. 

Considering that BDNF protein and mRNA levels are decreased in the ventral hippocampus of SERT^−/−^ rats [25,26,27,43], we sought to investigate whether BDNF gene overexpression in the ventral hippocampus was able to restore BDNF levels and anxiety- and depression-like behavior in SERT^−/−^ rats. While BDNF gene expression generates at least nine different transcripts [44], we targeted the overexpression of transcript IV because it is most downregulated in SERT^−/−^ rats [25]. Moreover, BDNF IV was selected due to its activity-dependent expression, association with depressive-like phenotypes [45] and its higher contribution to total levels of BDNF protein in the hippocampus [46]. The way a given genetic variant affects the function of a regulatory element remains poorly understood; however, it is known that untranslated regions are considered to influence gene expression by modulating mRNA stability and/or translational efficiency [47,48]. Moreover, it is known that individual transcript variants might differentially impact the complexity of apical and basal arbors of dendrites in vivo. BDNF II and VI, for example, significantly contribute to dendrite morphology in both CA1 and CA3 hippocampal neurons [49]. Accordingly, we hypothesize that overexpression of BDNF IV could directly or indirectly regulate the gene expression of the other BDNF transcripts. Therefore, in the first experiment we aimed to analyze the temporal dynamics of BDNF IV overexpression at one, two and four weeks following lentivirus infusion in the ventral hippocampus (vHIP) of SERT^+/+^ rats. Additionally, besides measuring BDNF IV levels, we also measured mRNA levels of total BDNF and BDNF VI through RT-qPCR analysis. Moreover, because the medial prefrontal cortex (mPFC) receives direct projections from the ventral hippocampus [50] and BDNF levels are known to be decreased in the PFC of SERT^−/−^ rats [25], we decided to measure BDNF levels in the prelimbic and infralimbic cortices as well. Thereafter, in a second experiment, following the viral infusions, SERT^−/−^ and SERT^+/+^ rats were submitted to behavioral testing. Behavior experiments included: (1) The sucrose consumption test, in which we assessed the rats’ preference for sucrose over water and the total sucrose intake in grams; (2) the forced swim test, during which we scored time spent on immobility, mobility and strong/high mobility; (3) the open field test, in which we evaluated distance moved, velocity and time and frequency in the center under novelty conditions; (4) the social interaction test, used to measure social and non-social behaviors such as self-grooming, allogrooming and nose contact, cage exploration and following; and (5) the HPA-axis reactivity test, a test where the response of the HPA-axis upon acute restraint stress was checked through measurement of plasma corticosterone (CORT) levels. 

## 2. Results

### 2.1. Lentivirus Transfection Leads to BDNF Overexpression in Ventral Hippocampus of SERT^+/+^ Rats Subsection

Molecular analyses were performed to examine the feasibility of BDNF lentivirus overexpressing exon IV in the ventral hippocampus (vHIP) of naïve SERT^+/+^ rats. To check temporal dynamics, mRNA levels were evaluated one, two and four weeks following BDNF or GFP lentivirus infusion. Due to the complexity of the BDNF gene and the distribution of its diverse transcripts variants across the brain [44], RT-qPCR was performed to measure total BDNF, BDNF IV and BDNF VI. Additionally, mRNA levels were measured not only in the vHIP but also in areas of high connectivity with the vHIP that are likewise involved in mood disorders, namely the prelimbic (PrL) and infralimbic (IL) cortex [14,51,52]. The results from each brain area are detailed below.

### 2.2. Local BDNF IV Lentivirus Infusion Leads to Overall BDNF Overexpression in the Ventral Hippocampus

**Figure 1:**
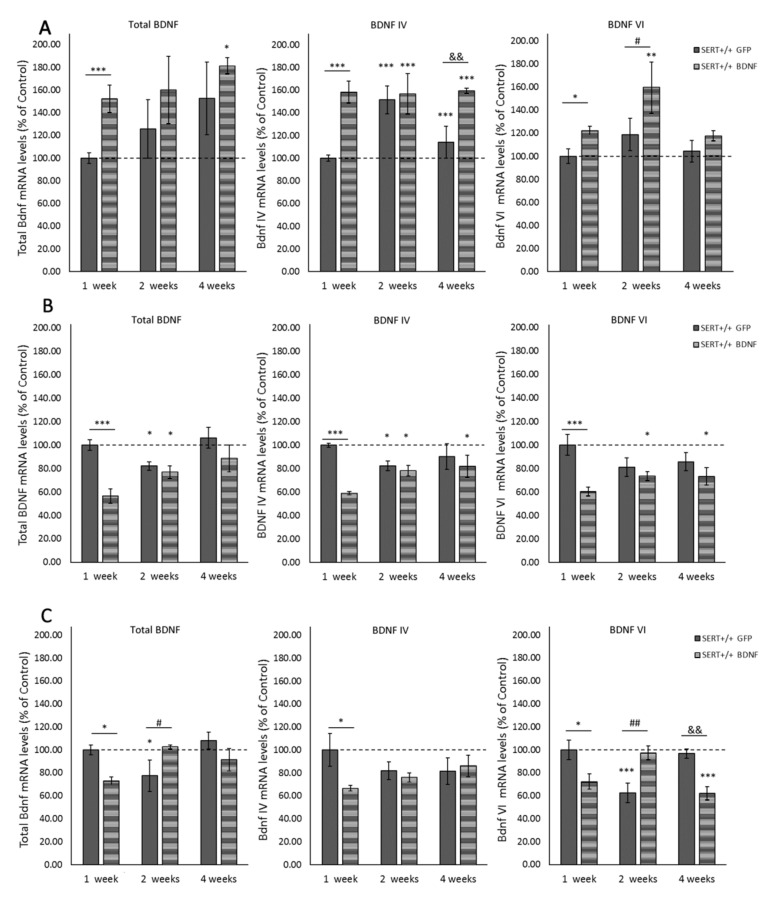
Modulation of total BDNF, BDNF IV and BDNF IV transcripts expression in SERT ^+/+^ animals infused with either GFP control or BDNF viral particles. BDNF mRNA levels were measured in the (**A**) ventral hippocampus, (**B**) infralimbic cortex and (**C**) prelimbic cortex one, two and four weeks after stereotaxic surgery. Data are expressed as fold change compared to the GFP-treated animals (set at 100%) and reflect mean ± SEM from 5–7 independent determinations. * = *p* < 0.05, ** = *p* < 0.01 and *** = *p* < 0.001 vs. GFP-1 week; # = *p* < 0.05 and ## = *p* < 0.01 vs. GFP-2 weeks; && = *p* < 0.01 vs. GFP-4 weeks.

Two-way ANOVA revealed significant main effects for the diverse BDNF transcripts analyzed. For example, a treatment main effect was observed for total BDNF (F_(1, 27)_ = 5.451, *p* = 0.027), BDNF IV (F_(1, 26)_ = 19.077, *p* < 0.001) and BDNF VI (F_(1, 27)_ = 8.389, *p* = 0.007; Figure 1A). Moreover, an interaction between treatment and time was found for BDNF IV (F_(2, 26)_ = 4.533, *p* = 0.02). PLSD post-hoc analysis revealed that BDNF IV lentivirus infusion led to an expected upregulation of this transcript in the vHIP. BDNF IV levels in the site of the injection (vHIP) were significantly increased in SERT^+/+^ BDNF-treated animals compared to the control GFP-treated rats, one (*p* < 0.001), two (*p* < 0.001) and four weeks (*p* < 0.001) after surgery. Interestingly, two weeks after the viral infusion, we also identified a higher gene expression of transcript IV in control-treated SERT^+/+^ rats (vs. one-week GFP-treated animals, *p* < 0.001). Furthermore, as shown in Figure 1A, pairwise comparisons also indicated an increase in total BNDF in SERT^+/+^ BDNF-treated rats one (*p* = 0.003) and for weeks after surgery (*p* = 0.032) in comparison to one-week GFP-treated animals. Additionally, BDNF VI levels were increased one (vs. one-week SERT^+/+^ GFP, *p* = 0.02) and two weeks after the surgery compared to controls (vs. one-week SERT^+/+^ GFP, *p* = 0.002; vs. two weeks SERT^+/+^ GFP, *p* = 0.031). In conclusion, BDNF lentivirus infusion in the vHIP caused local overexpression of BDNF IV mRNA, which was stable for at least four weeks. Additionally, it led to total BDNF and BDNF VI upregulation in the vHIP.

### 2.3. BDNF Overexpression in the vHIP Leads to BDNF mRNA Downregulation in the Infralimbic Cortex

As shown in Figure 1B, a significant reduction in the analyzed BDNF transcripts in the IL was observed following the transfection of BDNF lentivirus into the vHIP. Two-way ANOVA was computed and results demonstrated main effects for treatment (F_(1, 28)_ = 16.756, *p* < 0.001), time (F_(2, 28_) = 4.170, *p* = 0.026) and treatment vs. time interaction (F_(2, 28)_ = 4.903, *p* = 0.015) for total BDNF levels. Additionally, treatment main effects were observed for BDNF IV (F_(1, 27)_ = 12.515, *p* = 0.001) and BDNF VI (F_(1, 29)_ = 12.514, *p* = 0.001). There was also a treatment vs. time interaction (F_(2, 27)_ = 5.610, *p* = 0.009) for BDNF IV. Interestingly, as it can be seen in Figure 1B, pairwise comparisons revealed that one week following the infusion in the vHIP, the levels of BDNF in the IL of BDNF-treated SERT^+/+^ rats dropped about 40% (*p* < 0.001) compared to control-treated animals at the same time point. Notably, both BDNF- and control-treated groups presented decreased levels of BDNF in the second week after the surgery (*p* = 0.05). This decrease was sustained for at least four weeks in BDNF-treated animals for BDNF IV (*p* = 0.038 vs. one-week SERT^+/+^ GFP-treated) and BDNF VI levels (*p* = 0.01 vs. one-week SERT^+/+^ GFP-treated), but total BDNF levels were back to normal four-weeks after the virus infusion. In conclusion, we observed that BDNF lentivirus infusion in the vHIP altered BDNF levels in the infralimbic cortex, especially causing a significant decrease in BDNF mRNA expression one week after surgery.

### 2.4. BDNF Overexpression in the vHIP Alters BDNF Expression in the Prelimbic Cortex

As in the IL, the levels of BDNF were decreased in the PrL of BDNF-treated SERT^+/+^ one week after the viral infusion in the vHIP (Figure 1C). Specifically, main effects for the interaction between genotype and time point were found for total BDNF (F_(2, 28)_ = 6.044, *p* = 0.007) and BDNF VI (F_(2, 28)_ = 13.802, *p* < 0.001). Post-hoc analysis revealed that infusion of BDNF IV lentivirus particularly disturbed BDNF expression in a time- and transcript-dependent manner. For example, at the one-week time point, all the transcripts analyzed were decreased in the SERT^+/+^ rats treated with BDNF in comparison to controls (total BDNF *p* = 0.038, BDNF IV *p* = 0.02 and BDNF VI *p* = 0.014). However, as it is seen in Figure 1C, two weeks following the surgery, while control rats presented decreased levels of total BDNF and BDNF VI (respectively *p* = 0.024 and *p* < 0.001 vs. one-week SERT^+/+^ GFP-treated), SERT^+/+^ rats treated with BDNF had these transcripts levels normalized to the control levels. Moreover, four weeks following the surgery, particularly the levels of BDNF VI were decreased in the BDNF-treated animals (*p* < 0.001 vs. one-week SERT^+/+^ GFP-treated, *p* = 0.003 vs. four weeks SERT^+/+^ GFP-treated). In short, BDNF overexpression in the vHIP caused decreased BDNF levels in the PrL one week after infusion; however, two and four weeks after, it especially altered the expression of BDNF VI resulting in both increased levels two weeks following surgery and decreased levels four weeks after surgery.

### 2.5. Sucrose Consumption Test

Anhedonia is marked by a reduced interest in pleasurable events and it is present in depression. This depression-like symptom can be identified in rodents through a decrease in sucrose consumption, which can be accessed through the sucrose consumption test. Animals were exposed to two days of free access to a sucrose 8% solution. The results of the preference for sucrose above the water and the sucrose intake in grams are described below.

#### 2.5.1. Sucrose Preference: BDNF Upregulation Meliorates Anhedonia-Like Behavior in SERT^−/−^ Rats upon First Exposure to the Sucrose Solution

**Figure 2:**
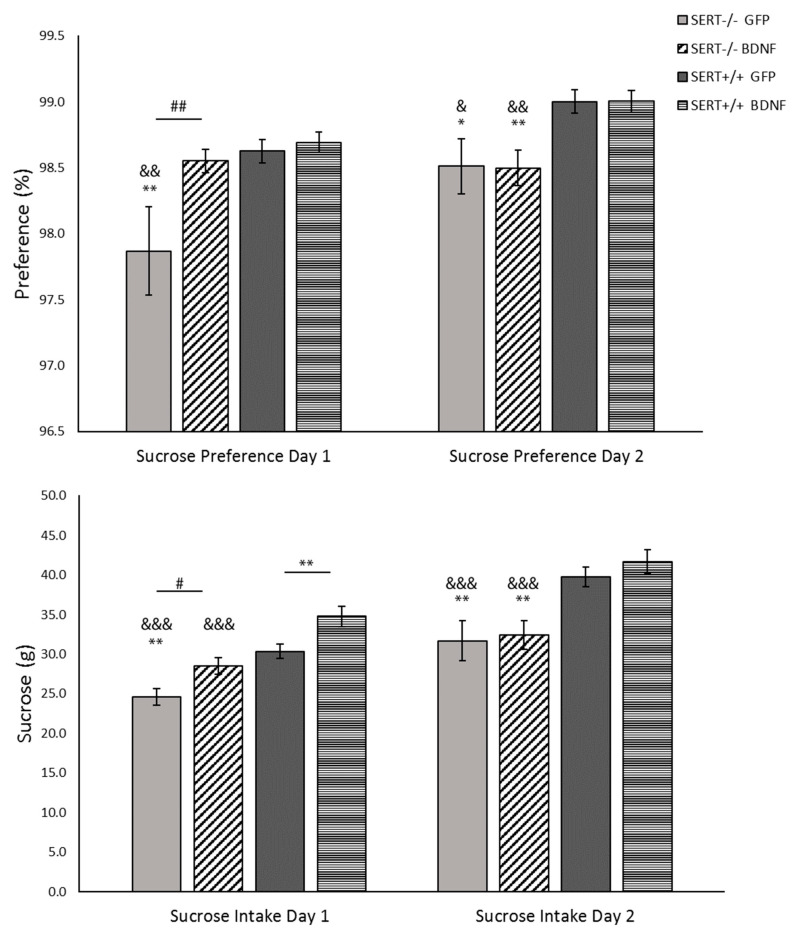
Sucrose consumption of 8% sucrose solution by SERT^−/−^ and SERT^+/+^ rats. Data are expressed as mean S.E.M. sucrose preference (sucrose intake/total fluid intake ×100%) and as mean S.E.M. total sucrose intake (g) per body weight (*n* = 10–12; * = *p* < 0.05 and ** = *p* < 0.01 vs. SERT^+/+^-GFP; & = *p* < 0.05, && = *p* < 0.01 and &&& = *p* < 0.001 vs. SERT^+/+^-BDNF; # = *p* < 0.05 vs. SERT^−/−^-GFP).

Preference for sucrose was analyzed in two sessions. In the first day of test, two-way ANOVA indicated genotype as well treatment main effects (F_(1, 36)_ = 6.980, *p* = 0.012 and F_(1, 36)_ = 4.854, *p* = 0.034, respectively). In detail, as shown in Figure 2, SERT^−/−^ rats treated with control virus presented a significant reduction in sucrose preference compared to both SERT^+/+^ controls (*p* < 0.01) and BDNF-treated SERT^−/−^ rats (*p* = 0.006). Meanwhile, the BDNF-treated SERT^−/−^ rats displayed similar sucrose preference compared to control-treated SERT^+/+^ animals. On the second day, the BDNF treatment effect in SERT^−/−^ was not present anymore and only a genotype effect was found (F_(1, 38)_ = 13.686, *p* < 0.001). Pairwise analysis indicated that both control- and BDNF-treated SERT^+/+^ had a higher preference for sucrose than both SERT^−/−^ groups (*p* < 0.05). In conclusion, as previously demonstrated, SERT^−/−^ rats displayed a lower preference for sucrose than SERT^+/+^ rats (Olivier et al., 2008). We found that BDNF treatment improved the preference for sucrose in SERT^−/−^ rats at least in the first day of the test.

#### 2.5.2. Sucrose Intake: BDNF Overexpression Exclusively Modulates Sucrose Intake on the First Day of the Sucrose Consumption Test 

As done for the sucrose preference, sucrose intake was measured in two sessions. The results reveal that, on the first day of the test, BDNF lentivirus treatment altered the consumption of a sweet solution in both SERT^+/+^ and SERT^−/−^ rats. This effect was lost on the second day (Figure 2). Two-away ANOVA showed a genotype (F_(1, 38)_ = 29.313, *p* < 0.001) and a treatment effect (F_(1, 38)_ = 14.198, *p* < 0.001) on the first day of the test. In detail, both SERT^−/−^ and SERT^+/+^ rats treated with BDNF presented a significantly higher intake of sucrose than their respective GFP-treated controls (BDNF- vs. GFP- SERT^−/−^, *p* = 0.017; and BDNF- vs. GFP- SERT^+/+^, *p* = 0.007). Moreover, compared to control SERT^+/+^ rats, only the GFP-treated SERT^−/−^ rats presented a lower intake of sucrose (*p* = 0.001). On the second day, just a genotype main effect was observed (F_(1, 38)_ = 23.280, *p* < 0.001). Post-hoc comparisons showed that BDNF treatment did not influence the intake in the second day and that—as previously described [29]—SERT ^−/−^ rats consumed less sucrose than the SERT^+/+^ rats (*p* < 0.01). Taken together, the sucrose intake was influenced by the BDNF overexpression, with a positive effect on the anhedonia-like behavior of SERT^−/−^ rats, leading to an even higher sucrose intake in SERT^+/+^ rats. However, this effect was only present on the first day of the test. Upon the second exposition to the sucrose bottles, the SERT^−/−^ anhedonia-like phenotype returned. 

### 2.6. BDNF Overexpression Decreases Immobility in the Forced Swim Test in SERT^−/−^ Rats

**Figure 3:**
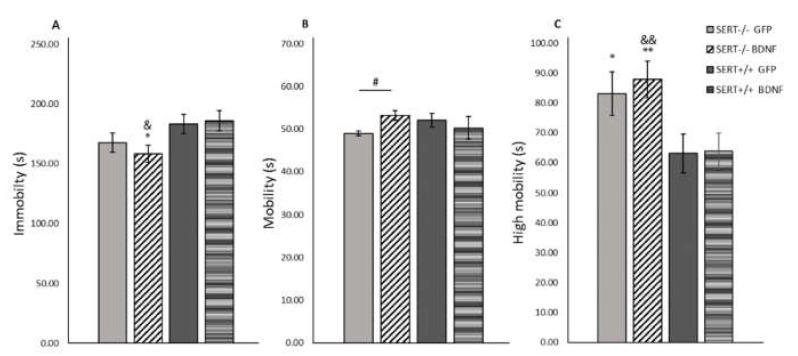
Mean (± SEM) measure of (**A**) immobility, (**B**) mobility and (**C**) high mobility in the forced swim test (FST). n = 10–12 rats per group. * = *p* < 0.05 and ** = *p* < 0.01 vs. SERT^+/+^ GFP; & = *p* < 0.05 and && = *p* < 0.01 vs. SERT^+/+^ GFP; # = *p* < 0.05 vs. SERT^−/−^ GFP). Two-way ANOVA, Fisher LSD post-hoc test.

When rodents are exposed to an inescapable stressor such as in the forced swim test, their motivation to cope with stress can be quantified by the percentage of time spent on immobility (behavioral passivity) or performing a highly mobile (escape-like) behavior [53]. Two-way ANOVA determined that for both extreme swimming modalities, immobility and high mobility, genotype main effects were found (F_(1, 37)_ = 7.589, *p* = 0.009; and F_(1, 37)_ = 11.237, *p* = 0.002, respectively), while no main effects were found for normal swimming (mobility) behavior (Figure 3). Moreover, pairwise comparisons demonstrated that SERT^−/−^ rats were much more prompted to develop an escape-like behavior in comparison to SERT^+/+^ rats (*p* < 0.05). Furthermore, as shown in Figure 3A, we observed that BDNF upregulation in the SERT^−/−^ rats resulted in a decreased immobility in comparison to both SERT^+/+^ groups (vs. SERT^+/+^ GFP, *p* = 0.025 and vs. SERT^+/+^ BDNF, *p* = 0.011). In conclusion, BDNF overexpression did not decrease the anxiety-like behavior in SERT^−/−^ rats expressed by increased time spent in the high mobility swimming modality; on the other hand, BDNF upregulation in the vHIP affected behavioral passivity, as expressed by a decrease in time spent on immobility in SERT^−/−^ rats.

### 2.7. BDNF Upregulation Decreases Anxiety-Like Behavior in SERT^−/−^ Rats in the Open Field Test

**Figure 4:**
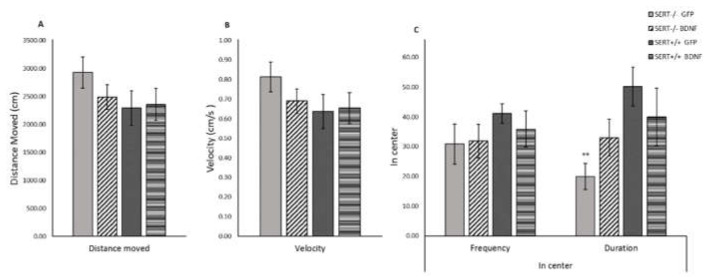
Novelty-induced locomotor activity expressed as mean (± SEM) (**A**) distance moved, (**B**) velocity and (**C**) frequency and time spent in center. *n* = 10–12; ** = *p* < 0.01 vs. SERT^+/+^ GFP). Two-way ANOVA, Fisher LSD post-hoc test.

Rodents may present a higher activity when they are introduced to a new environment [54]. A decrease in central locomotion (frequency and time spent in the central part of the arena), together with a general decrease in the locomotion (distance moved and velocity) can be interpreted as an anxiogenic-like behavior [55]. As previously reported [56], no differences between SERT^−/−^ and SERT^+/+^ rats were observed for the distance moved or velocity (Figure 4). However, we did observe a genotype main effect for time spent in the center of the test-cage (F_(1, 37)_ = 6.557, *p* = 0.015, Figure 4C). Further post-hoc analysis revealed that control-treated SERT^−/−^ rats spent less time in the center of the cage than control SERT^+/+^ rats (*p* = 0.007); meanwhile, BDNF injected SERT^−/−^ rats spent similar time in the center compared to the SERT^+/+^ rats. In conclusion, no alterations in the locomotor distance or speed were observed due to treatment or genotype. In contrast, the duration these animals spent in the center of the novel environment highlighted the behavioral differences between SERT^−/−^ and SERT^+/+^ rats. BDNF upregulation in SERT^−/−^ rats normalized the level of anxiety to that of SERT^+/+^ rats. 

### 2.8. BDNF in the Social Interaction Test

**Figure 5:**
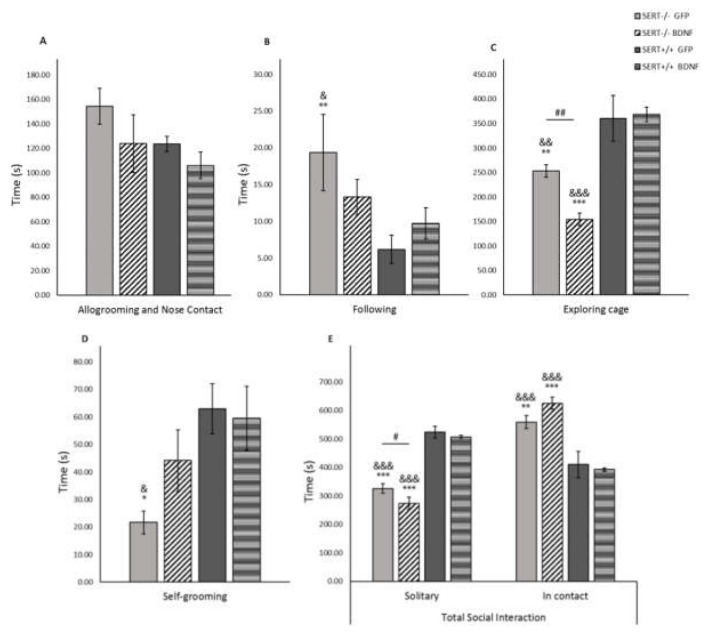
Social interaction test expressed as mean (± SEM) of the time spent in (**A**) allogroming and nose contact, (**B**) following, (**C**) exploring cage, (**D**) self-grooming and (**E**) total social contact and solitary behaviors. *n* = 10–12; * = *p* < 0.05, ** = *p* < 0.01 and *** = *p* < 0.001 vs. SERT^+/+^ GFP; & = *p* < 0.05, && = *p* < 0.01 and &&& = *p* < 0.001 vs. SERT^+/+^ BDNF; # = *p* < 0.05 vs. SERT^−/−^ GFP). Two-way ANOVA, Fisher LSD post-hoc test.

The social interaction test was first introduced by File and Hyde in 1978 [57]. In humans, impaired social interaction is considered a core symptom in depressive disorder [58,59]. In our experimental conditions, as demonstrated in Figure 5, we observed a marked genotype influence on the general time spent in both contact and solitary behaviors. Specifically, SERT^−/−^ animals tended to spend more time in contact with the test partner than SERT^+/+^ rats. For solitary behavior, namely the time the animal spent performing individual actions such as self-grooming or cage exploration, a significant main genotype effect was found F_(1, 36)_ = 147.868, *p* < 0.001. Further posthoc analysis have shown that both GFP and BDNF-treated SERT^−/−^ rats presented decreased solitary time when compared to both GFP and BDNF-treated SERT^+/+^ animals (*p* < 0.001). Moreover, BDNF treatment in SERT^−/−^ rats reduced solitary behavior when compared to control-treated SERT^−/−^ rats (*p* = 0.041). An opposite effect was observed in the time animals spent in contact. Two-way ANOVA revealed a significant genotype main effect F_(1, 36)_ = 50.134, *p* < 0.001 in the time that test animals were standing still in close contact with their test partner or performing behaviors such as allogrooming and nose contact. Pairwise comparisons showed that SERT^−/−^ rats, independently of the treatment received, spent more time in contact with their test-partner than SERT^+/+^ rats (*p* < 0.01). Concerning the interaction between the animals while in contact with each other, no statistically significant differences in the allogrooming and nose contact behaviors were found. However, main genotype effects were observed in the following, self-grooming and exploring cage non-contact behaviors (F_(1, 33)_ = 6.785, *p* = 0.014; F_(1, 32)_ = 7.480, *p* = 0.01; and F_(1, 36)_ = 43.600, *p* < 0.001, respectively). Additionally, a significant genotype and treatment interaction was observed in the cage exploratory behavior (F_(1, 36)_=43.600, *p* < 0.001. Interestingly, control treated SERT^−/−^ rats presented increased following behavior when compared to SERT^+/+^ animals (*p* = 0.009 vs. GFP SERT^+/+^ rats and *p* = 0.043 vs. BDNF SERT^+/+^), while BDNF-treated SERT^−/−^ animals did not differ from wild type animals. Likewise, while control-treated SERT^−/−^ rats presented decreased self-grooming behavior compared to GFP and BDNF SERT^+/+^ rats (*p* < 0.05), BDNF treated SERT^−/−^ rats presented self-grooming behavior at the level of SERT^+/+^ rats. Exploratory behavior was also affected by genotype. We observed that SERT^−/−^ rats presented less cage exploration than SERT^+/+^ rats in all treatment groups (*p* < 0.01). Moreover, BDNF treatment in SERT^−/−^ rats induced a significant decrease in exploring the test cage when compared to GFP treated SERT^−/−^ animals (*p* = 0.006). In summary, BDNF treatment did not affect social interaction in SERT^+/+^ animals, while it increased self-grooming and decreased following, solitary and exploratory behaviors in SERT^−/−^ rats compared to GFP-treated SERT^−/−^ rats. Moreover, in general, irrespective of the treatment, SERT^−/−^ rats generally spent less time solitary and more time in social contact and consequently less time in exploratory behavior than SERT^+/+^ animals.

### 2.9. BDNF Overexpression Reduces CORT Levels in SERT^−/−^ Rats in the HPA-Axis Reactivity Test

**Figure 6:**
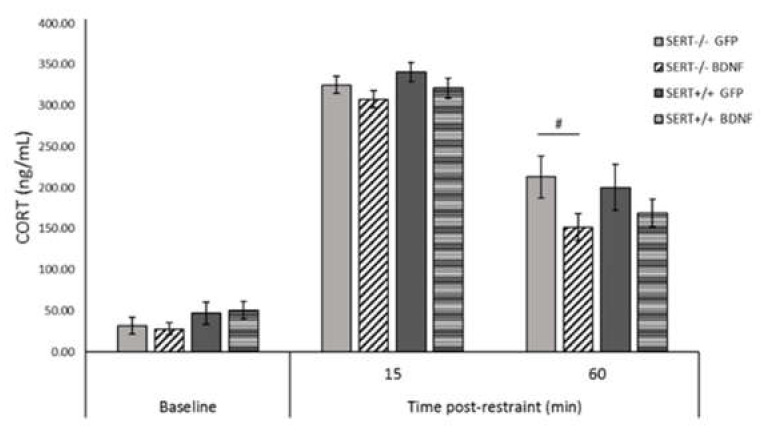
HPA-axis reactivity assessment. Corticosterone (CORT) levels are expressed mean ± (SEM) of plasma CORT levels 5 min before restraint stress (baseline) and 15 and 60 min post-restraint stress. *n* = 10–12; # = *p* < 0.05 vs. SERT^−/−^ GFP. Linear mixed model.

Acute restraint stress can activate the hypothalamic-pituitary-adrenal axis (HPA-axis), resulting in the release of CORT in rodents, which can be a measure of the (mal) functionality of the HPA-axis. The HPA-axis response to stress was evaluated 15 and 60 min after restraint stress (Figure 6). A linear mixed-effect analysis was conducted and no interactions were found. However, pairwise comparisons indicated a significant decrease in CORT levels of BDNF-treated SERT^−/−^ rats 60 min after acute stress (*p* = 0.014), indicating that BDNF overexpression in the ventral hippocampus likely reduced the HPA reactivity in the SERT^−/−^ rats at this timepoint. No differences in the baseline CORT levels were found. Conclusively, while no basal or stress-induced differences in CORT levels were found between control-treated SERT^−/−^ and SERT^+/+^ rats, BDNF upregulation induced a decrease in the CORT levels in SERT^−/−^ rats versus control-treated SERT^−/−^ rats. 

## 3. Discussion

For many years, it has been hypothesized that the intricate system regulating the BDNF gene results in the generation of BDNF transcripts that create a spatial code for BDNF gene expression throughout the brain [60]. Definitive conclusions regarding the role different transcripts play in BDNF signaling are still lacking, but it is of great interest giving that BDNF mRNA variants are differently (down)regulated in individuals suffering from neuropsychiatric disorders, including mood disorders. Considering this, we aimed to investigate the effects of BDNF IV overexpression in the ventral hippocampus of animals presenting both depressive-like behavior and BDNF downregulation. Before checking for the behavioral outcome, we checked for the BDNF overexpression itself following BDNF local brain infusion in the ventral hippocampus. Here, we report that lentivirus BDNF IV infusion into the vHIP caused a significant overexpression of BDNF in the vHIP, followed by upregulation of other BDNF transcripts, such as total and BDNF VI levels. Interestingly, we observed that the upregulation of BDNF in the vHIP disturbed the BDNF gene expression in other brain areas. Particularly, we examined the PrL and IL and noticed an accentuated decrease in BDNF expression in these areas, especially in the first week following the BDNF viral infusion. Furthermore, we demonstrated that BDNF overexpression resulted in task- and genotype-dependent modifications in the phenotypic outcome from SERT^−/−^ and SERT^+/+^ rats. For instance, in the sucrose consumption test, BDNF treatment improved the preference for sucrose in SERT^−/−^ rats and increased sucrose intake in SERT^−/−^ and SERT^+/+^ rats, but only in the first day of the test. Additionally, BDNF-infused SERT^−/−^ rats presented decreased immobility in the forced swim test and lower anxiety levels in the novelty-induced locomotor activity test compared to controls. Moreover, BDNF upregulation in SERT^−/−^ rats regulated some of the observed behaviors in relation to SERT^+/+^ rats. For instance, it decreased following behavior and increased self-grooming to the level of SERT^+/+^ rats. BDNF infusion in SERT^−/−^ rats also decreased solitary behavior and increased passive interaction with test partner. Finally, BDNF upregulation induced a decrease in HPA-axis activity, reflected by the decreased CORT levels in SERT^−/−^ rats.

Precise time-, brain region- and stimuli-dependent BDNF production might be mediated by the expression of multiple BDNF mRNA variants [46]. At least nine promoters are involved in the regulation of BDNF [44]; among them, promoter IV is an activity-dependent gene, leading to the expression of BDNF upon neuronal activity [61]. Interestingly, molecular analysis of the BDNF IV overexpression in this study revealed exciting features regarding the BDNF IV effect over other BDNF transcripts and regarding the vHIP connection with frontal areas of the rat brain. In short, one week after the viral infusion into the vHIP, we demonstrated that total BDNF, BDNF IV and BDNF VI were overexpressed in the vHIP and downregulated in the PrL and IL of BDNF-infused SERT^+/+^ rats. In the IL, the decreased BDNF levels of variants IV and VI were stable for at least four weeks, showing a continuous influence from the vHIP BDNF upregulation. On the other hand, in the PrL, we noticed a time-dependent regulation specific to total and BDNF VI. Two weeks after BDNF infusion, PrL levels of total BDNF and BDNF VI were higher in BDNF-infused animals, but in the fourth week, BDNF VI levels were again downregulated. The medial prefrontal cortex (mPFC) receives direct projections from the ventral hippocampus [50]. Studies in cultured hippocampal neurons revealed activity-induced BDNF dendritic release [8,11,62]. Accordingly, these data indicate that, likely, BDNF upregulation in the vHIP caused negative feedback in the BDNF transcription of downstream targets such as the PrL and IL. Importantly, just as the vHIP-mPFC synchrony was shown to be modulated by negative emotions like anxiety [63], vHIP to mPFC transcriptional control might likewise induce effects on emotional outcome. Furthermore, the different effects in PrL and IL BDNF gene expression might specifically modulate cognitive (mal)functions, considering that these two mPFC subdivisions have different projections sites [64,65].

Anhedonia is a core symptom in mood disorders [66]. This negative emotional state has previously been demonstrated in SERT^−/−^ rats [29] and was replicated in this study. In our experimental conditions, control-treated SERT^−/−^ rats displayed anhedonia-like behavior when compared to controls SERT^+/+^ rats. This anhedonia-like behavior was suppressed in the SERT^−/−^ rats following BDNF upregulation. However, the enhancement in sucrose preference and sucrose intake was limited to the first day of the test and it was lost on the second day of the test. This means that animals preferred the sucrose solution only on the first day of the test and presented decreased sucrose preference and intake on the second day of the test. Therefore, the upregulation of BDNF IV in the vHIP likely ameliorated the anhedonia-like symptoms in SERT^−/−^ rats only when the novelty factor was present. After habituation to the novel sweet taste, the anhedonic phenotype was prevalent in SERT^−/−^ animals in comparison to SERT^+/+^ rats. 

Another core endophenotype in mood disorders is behavioral despair [28]. In rodents, this symptom can be evaluated through the forced swim test [67]. In the present study we did not observe differences in immobility scores when comparing control-treated SERT^−/−^ to SERT^+/+^ rats. This result is at odds with previous studies using SERT^−/−^ rats and mice, in which immobility was increased in knockout animals [29,68]. It is possible that stress associated with the brain surgery, as well as IVC cage housing, contributed to the different outcome in the present study. Interestingly, BDNF upregulation in the vHIP decreased immobility time in SERT^−/−^ rats in comparison to GFP-treated SERT^−/−^ animals, suggesting that BDNF overexpression contributed to an adaptive learned response [69]. In line with this finding, Karpova et al. [70] demonstrated that an increase in hippocampal BDNF IV caused by postnatal SSRI exposure was associated with decreased immobility in the forced swim test. Further, we demonstrated that SERT^−/−^ rats, independently of the treatment, displayed higher escape-behavior than SERT^+/+^ rats. Increased strong mobility in the forced swim test might indicate enhanced anxiety-like behavior displayed by SERT^−/−^ rats. Anxiety-like behavior in SERT^−/−^ rats might be due to changes in the functioning of the GABAergic system in SERT^−/−^ rats [27,43,71,72], causing GABA downregulation and consequently, increased anxiety-like behavior [73,74]. 

In the novelty-induced locomotor activity test, we found that SERT^−/−^ rats spent less time in the center of the test-cage than SERT^+/+^ rats, again pointing to increased anxiety-like behavior [29]. Interestingly, SERT^−/−^ rats receiving BDNF virus as treatment did not differ from SERT^+/+^ controls, indirectly suggesting that BDNF upregulation likely decreased the SERT^−/−^ rats’ anxiety-like behavior in this behavior paradigm. This BDNF anxiolytic-like action modulated by exposition to novelty is in agreement with the results from the sucrose consumption test, in which SERT^−/−^ rats seems to present enhanced negative emotionality in connection to the novel taste and this behavior was reduced by BDNF IV overexpression in the vHIP. Additionally, in our experimental conditions, we replicated the previous observation that no alterations in the locomotor distance or speed were observed in SERT^−/−^ rats in comparison to SERT^+/+^ rats [56,75]. This also indicates that the decreased immobility and increased high mobility observed in SERT^−/−^ rats in the forced swim test was not due to increased locomotor activity.

Following the open field test, the rats were submitted to the social interaction test in the same apparatus to which the animals have been exposed and familiarized with. In accordance with previous findings, SERT^−/−^ rats presented an increased following or chasing behavior, indicating that these animals are actively looking for social contact [76,77]. Interestingly, BDNF infusion in SERT^−/−^ rats decreased the following behavior to the level of the observed behavior in SERT^+/+^ rats. Moreover, in previous studies SERT^−/−^ rats presented decreased active/playful social behaviors such as allogrooming and nose contact; however, in our experimental conditions, we did not find significant genotype differences in such playful behaviors. Possibly, the different behavior found in the current study might be explained by the age of the tested animals, as social play behavior is most abundantly observed in younger animals [76]. Additionally, in contrast to previous findings [77,78], SERT^−/−^ rats spent less time in solitary behavior and less time in self-grooming behavior. Usually, as a response to environmental changes rodents present increased self-grooming. This behavior might be used to restore stress-induced disturbed homeostatic status [79,80,81,82] It is possible that lower self-grooming in control treated SERT^−/−^ rats and increased contact with test-partner are an indicative that these animals were more inclined to a passive prosocial behavior, especially also because allogrooming and nose-to-nose contact were not altered by BBNF infusion (active social behaviour) were not affected by SERT genotype. BDNF infusion in SERT^−/−^ rats reversed the decrease in self-grooming and contributed even more to a decrease in solitary behavior, which could further increase passive social interaction. 

Adaptive responses to stressors require behavioral flexibility that depends on a plastic brain. Considering this, we also investigated the effects of hippocampal upregulation of BDNF IV on HPA-axis activity through the measurement of corticosterone (CORT) levels following acute stress. We demonstrated that 60 min after the acute stress, BDNF upregulation in the vHIP caused a significant decrease in CORT levels in SERT^−/−^ rats compared to GFP-treated SERT^−/−^ rats, indicating that BDNF overexpression affected the decreasing phase in CORT levels [83] by enhancing the negative feedback over the HPA-axis. Regarding the peak in CORT levels, at 15 min following the acute restraint stress, we observed that CORT levels were not affected by BDNF upregulation or genotype. Likewise, basal CORT levels were comparable in SERT^−/−^ and SERT^+/+^ rats, denoting a lack of BDNF modulation in baseline levels. In agreement with these results, studies in SERT^−/−^ mice also have shown that plasma basal CORT levels [84,85,86] or stress-response CORT levels [87] were not altered in comparison to SERT^+/+^ mice. However, in contrast with our results, van der Doelen et al., (2014) [88] have shown that non-stressed SERT^−/−^ rats presented increased baseline levels of CORT compared to SERT^+/+^ rats, whereas when submitted to early life stress, SERT^−/−^ rats present lower levels of CORT than early-life-stressed SERT^+/+^ rats. In our experimental conditions, the SERT^−/−^ rats were not stressed in their early life stage; however, they underwent surgery stress, isolation stress (in the sucrose consumption test) and were exposed to a novel environment in the locomotor activity test. Therefore, although animals were given time to recover, it is possible that the sequence of behavioral tests shaped an adaptive CORT response to stress [89] as well as determined the differential CORT basal levels [88]. Altogether, these data suggest that BDNF affects the HPA-axis activity in a genotype and phase-dependent manner. 

Depression is a complex neuropsychiatric disorder comprising multiple neural circuit processes [90]. Animal models such as the SERT^−/−^ rat comprise a useful tool to study targeted circuits that are found in both rodents and humans. While complete function loss of the SERT cannot be found in humans, behavioral and biomolecular changes in SERT^−/−^ are comparable to short allele carriers of the human 5-HTTLPR [28]. In agreement with the neurotrophic hypothesis of depression, these animals present, under basal conditions, downregulation of BDNF mRNA (especially exon IV) and protein levels in the ventral hippocampus and prefrontal cortex [25,27]. Modulation of BDNF IV levels in the ventral hippocampus showed to have a positive effect on mood disorder-related symptoms. Selective upregulation of a specific BDNF exon can lead to specific neuronal network reinforcement [60]. However, further characterization of the effects that BDNF overexpression might exert over other brain areas targeted by the vHIP, such as the nucleus accumbens and amygdala, is still needed. In addition, given that BDNF did not decrease escape behavior in the forced swim test, indicative for anxiety-like behavior, it is still unclear which circuits might be involved in the forced swim test escape-behavior response. Moreover, it is uncertain whether BDNF overexpression in the vHIP restored the decreased GABAergic inhibitory regulation in SERT^−/−^ rats [27,71,72] Nonetheless, despite necessary further investigations, we hypothesize that BDNF IV upregulation in the vHIP might be a good candidate for modulation of emotional behaviour in the treatment of mood disorders. While performing stereotaxic surgery in humans does not seem to be a feasible treatment for mood disorders in humans, advances in pharmaceutical technology have shown that the delivery of drugs and genes into the brain through peripheral bloodstream route using non-viral liposomes is possible [91]. Therefore, the additional studies to understand the BDNF dynamics in the vHIP as well as other brain areas of SERT^−/−^ rats might help to elucidate whether it would be possible to develop specific gene target-delivery approaches, allowing a future novel, rational treatment to bring individualized and improved care for patients with mood disorders.

## 4. Materials and Methods

### 4.1. Animals

**Figure 7:**
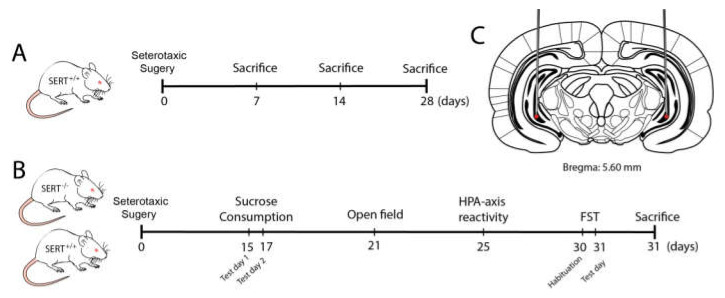
Schematic representation of the experimental design. (**A**) Evaluation of BDNF overexpression in naïve SERT^+/+^ rats one, two and four weeks following viral infusion. (**B**) Behavioral tests: Viral infusion followed by behavioral tests including sucrose consumption test, HPA-axis reactivity, open field and forced swim test (FST). (**C**) Representation of the local of the infusion of either BDNF or control virus.

SERT^−/−^ rats (Slc6a41Hubr) were generated by N-ethyl-N-nitrosourea (ENU)-induced mutagenesis on a Wistar background [92] and outcrossed with commercially available Wistar^CrI:WI^ rats obtained from Charles River Laboratories (Horst, the Netherlands) for at least 15 generations. Ear punches were taken at the age of 21 days for genotyping, which was done by LGC (Hoddesdon, UK). SERT^+/+^ rats were used to check BDNF virus overexpression in naïve animals (five to seven animals per group). For the behavioral experiments, 10 to 12 male SERT^−/−^ and wild-type (SERT^+/+^) rats per group, weighing 350–400 g at the beginning of the study, were used (see experimental design in Figure 7). All animals were housed in temperature-controlled rooms (21 °C) with standard 12/12-h day/night-cycle (lights on at 7:00 am) and food and water available ad libitum. Five to seven days before surgery, animals were socially housed in individually ventilated (IVC) cages for habituation. After surgery, animals were separately housed in the IVC cages until recovery. Animals were socially isolated during the sucrose consumption test; thereafter, the animals we socially housed again and kept under the same temperature and day/night-cycle throughout the entire experiment. All experiments were approved by the Central Committee on Animal Experiments (Centrale Commissie Dierproeven, CCD, The Hague, The Netherlands), and all efforts were made to reduce the number of animals used and their suffering.

### 4.2. Stereotaxic Surgery

Rats were anesthetized using isoflurane (5% induction, 2–3% maintenance). Lidocaine (10% m/v) was used for local anesthesia. Animals were fixed in a robot stereotaxic frame (StereoDrive, Neurostar, Germany). The coordinates for the site of the injection were theoretically determined based on the Paxinos & Watson (2007) rat brain atlas [93] and checked through histological evaluation of 30 µm brain slices from dye-infused SERT^+/+^ rats. The total volume of 2 μL of either BDNF lentivirus particles (transcript variant IV under CMV promoter, NM_001270633.1) or pLenti-C-mGFP control lentivirus particles, was bilaterally infused into the ventral hippocampus according to the following coordinates: AP −5.60 mm, ML ±5.0 mm, DV −7.6 mm. After surgery, animals were placed in IVC cages (Sealsafe Plus GR900 green line, Tecniplast, Italy) until sacrifice.

### 4.3. RNA Preparation and Gene Expression Analysis by Quantitative Real-Time PCR

Total RNA was isolated from the prelimbic and infralimbic region of the mPFC by single-step guanidinium isothiocyanate/phenol extraction using PureZol RNA isolation reagent (Bio-Rad Laboratories; Segrate, Italy), according to the manufacturer’s instructions, and then quantified by spectrophotometric analysis (NanoDrop™1000, Thermo Scientific, Waltham, MA, USA). Following total RNA extraction, an aliquot of each sample was treated with DNase to avoid DNA contamination. Then, the samples were processed for real-time PCR to assess total mRNA expression levels of BDNF, BDNF isoform IV and VI. The analyses were performed by TaqMan qRT–PCR instrument (CFX384 real-time system, Bio-Rad Laboratories S.r.l.) using the iScript one-step RT–PCR kit for probes (Bio-Rad Laboratories). Samples were run in 384-well formats in triplicates as multiplexed reactions with a normalizing internal control (36B4). Thermal cycling was initiated with incubation at 50 °C for 10 min (RNA retrotranscription) and then at 95 °C for 5 min (TaqMan polymerase activation). After this initial step, 39 cycles of PCR were performed. Each PCR cycle consisted of heating the samples at 95 °C for 10 s to enable the melting process and then for 30 s at 60 °C for the annealing and extension reactions. Data were analyzed with the comparative threshold cycle (ΔΔCt) method using 36B4 as a reference gene. Primers and probe for BDNF exon IV and VI were purchased from Life Technologies (BDNF exon IV: ID EF125679 and BDNF exon VI: ID EF125680). Primers and probe for total BDNF and 36B4 were purchased from Eurofins MWG-Operon. Their sequences are shown below:

total BDNF: Forward primer 5′-AAGTCTGCATTACATTCCTCGA-3′, reverse primer 5′-GTTTTCTGAAAGAGGGACAGTTTAT-3′, probe 5′-TGTGGTTTGTTGCCGTTGCCAAG-3′;

36B4: Forward primer 5′-TTCCCACTGGCTGAAAAGGT-3′, reverse primer 5′-CGCAGCCGCAAATGC-3′, probe 5′-AAGGCCTTCCTGGCC GATCCATC-3′.

### 4.4. Behavioral Tests

#### 4.4.1. Sucrose Consumption Test

After stereotaxic surgery, animals were housed individually and provided with two bottles of water for habituation. Before the test, side preference was checked for five days. The sucrose consumption test was adapted from Olivier et al., (2008) [29] and consisted of two days of free-choice access to 24 h sucrose versus water bottles with a water-only bottle choice in between the two days. In detail, on test day 1, one of the water bottles was replaced by a sucrose 8% solution and animals had free drinking access for a period of 24 h. Next, animals received water in both bottles for 24 h, ending with another 24 h of free choice between water and sucrose 8% solution on test day 2. The position of the bottles was switched from sucrose consumption test day 1 to the test day 2 to prevent spatial bias. Liquid intake and bodyweight were measured daily. The data are presented as the preference of sucrose above water (sucrose intake in ml divided by total intake × 100%) and the intake in grams of a 100% sucrose solution per kg bodyweight (intake in ml corrected for the voluminal weight of sucrose and recalculated toward a 100% solution divided by bodyweight in kg).

#### 4.4.2. HPA-Axis Reactivity Test

Hypothalamic–pituitary–adrenal (HPA) axis reactivity was assessed through the measurement of corticosterone levels in the plasma. Usually, when rodents are submitted to stress, plasma concentrations of corticosterone (CORT) peak after 15 to 30 min and gradually decrease 60 to 90 min later to the pre-stress levels (de Kloet et al., 2005). Therefore, blood samples from tail cuts were collected in capillary blood collection tubes (Microvette^®^ CB 300 Di-Kalium-EDTA, Sarstedt, Germany) 5 min before and 15 and 60 min after 30 min of restraint stress. Rodent restrainers Broome-style were used for the restraint stress (554-BSRR, Bio-services, The Netherlands). Blood samples were centrifuged (3400 rpm for 15 min at 4 °C) and the plasma was stored at −80 °C until analysis. CORT levels were measured using a liquid chromatography-tandem mass spectrometry (LC-MS/MS).

#### 4.4.3. Open Field Test

Novelty-induced locomotor activity was recorded by video recording in Phenotyper^®^ cages (Noldus Information Technology, Wageningen, The Netherlands). The cages (45 cm × 45 cm × 45 cm) were made of transparent Perspex walls and a black floor. Each cage had a top unit containing a built-in digital infrared-sensitive video camera, infrared lighting sources and hardware needed for video recording. To explore the novelty factor, animals were not exposed to this cage previously and the cages were cleaned with 70% alcohol solution between trials to prevent transmission of olfactory cues. Spontaneous locomotor activity was monitored for 1 h and the following parameters were scored using Ethovision XT 11.5 (Noldus Information Technology, Wageningen, Netherlands): Distance moved, velocity, frequency and time spent in the center of the cage [56,94].

#### 4.4.4. Social Interaction Test

Social interaction behavior was recorded by video recording in Phenotyper^®^ cages (Noldus Information Technology, Wageningen, The Netherlands). The cages (45 cm × 45 cm × 45 cm) were made of transparent Perspex walls and a black floor. Each cage had a top unit containing a built-in digital infrared-sensitive video camera, infrared lighting sources and hardware needed for video recording. Video recordings were used to score behavior from captured video files by using Observer XT 12.5 (Noldus Information Technology, Wageningen, The Netherlands). Social interaction pairs were designed such that both rats were genotype and treatment matched and that rats from the same home cage were not paired. Animals were tested in the same cage 48 h following the open field test to minimize any initial novelty-induced behavior by repeated exposure to the test environment. On the test day, test pairs were isolated for 3.5 h before the test to increase the amount of social behavior and were subsequently tested for 15 min. One observer blind to the test subjects’ genotype manually scored the time spent in the following social interaction behaviors: Allogrooming and nose contact—the focal animal has one or both front paws on top of the other and pulls/licks at its fur or sniffs, exploring the other animal; following/chasing—the focal animal moves/follow the other to maintain a close distance while the other animal is moving around/away; and self-grooming—forepaw licking, face washing, scratching, body grooming and genital grooming. Total contact time and solitary behavior patterns were also scored. Behavior was assessed per individual subject. Subjects were used only once. Test cages were cleaned with 70% ethanol between test subjects. 

#### 4.4.5. Forced Swim Test

The forced swimming test was performed as previously described [67]. Briefly, rats were individually placed in cylindrical glass tanks (50 cm height, 20 cm diameter) filled to a height of 30 cm with 23 ± 1 °C water. The test consisted of two sessions. In the first session, animals were submitted to a habituation period of 15 min, then 24 h later, to a second session of 5 min. The video recordings of the second session were used to automatically score the movements of the rats through a computerized system (Ethovision XT 10, Noldus, The Netherlands). Scored behaviors were ‘immobility’, which reflects no movement at all and/or minor movements necessary to keep the nose above the water; ‘mobility’, reflecting movement that corresponds to swimming activity; and ‘strong mobility’, reflecting ‘escape behavior’ (e.g., climbing against the walls and diving). Settings within Ethovision were adjusted based on manually recorded sessions (immobility/mobility threshold: 12; mobility/strong mobility threshold: 16.5 [95,96].

### 4.5. Statistical Analysis

The data were checked for outliers and normality (using the Shapiro–Wilk statistic) and extreme outliers were windsorized. Two-way analysis of variance (ANOVA) was computed for gene expression analysis, with time, genotype and treatment as independent factors. The outcomes of sucrose consumption, open field and forced swim tests and post-behavioral gene expression were also analyzed through Two-way ANOVA considering genotype and treatment as fixed factors. Post-hoc Fisher’s Protected Least Significant Difference (PLSD) or independent sample t-tests were performed where applicable to compare individual group differences. All these statistical analyses were carried out using IBM^®^ SPSS^®^ statistics, version 23 (IBM software, USA). Data from the HPA-axis reactivity test were subjected to a linear mixed model to account for repeated measurements and multiple factor analysis using the LME4 package in R (3.5.1). Time, genotype and treatment effects were modeled as a fixed effect, together with their pairwise double interactions and their triple interactions. Subject intercepts were modeled as random effects. A likelihood-test ratio was used to assess fixed effect significance. Post-hoc tests were performed with the multicomp package, which accounts for multiple hypothesis testing. Significance was accepted at a *p* < 0.05 threshold. Descriptive statistics are provided as mean +/− 1 standard error of the mean (SEM).

## Data Availability

Data is contained within the article.
